# Elevated IGF-1 with GH suppression after an oral glucose overload: incipient acromegaly or false-positive IGF-1?

**DOI:** 10.1590/2359-3997000000193

**Published:** 2016-08-23

**Authors:** Pedro W. Rosario, Maria R. Calsolari

**Affiliations:** 1 Santa Casa de Belo Horizonte Belo Horizonte MG Brazil Programa de Pós-Graduação e Serviço de Endocrinologia, Santa Casa de Belo Horizonte, Belo Horizonte, MG, Brazil

**Keywords:** Acromegaly, elevated IGF-1, GH suppression

## Abstract

**Objective:**

To report the evolution of patients with a suggestive clinical scenario and elevated serum insulin-like growth factor-1 (IGF-1), but growth hormone (GH) suppression in the oral glucose tolerance test (OGTT), in whom acromegaly was not initially excluded.

**Subjects and methods:**

Forty six patients with a suggestive clinical scenario, who had elevated IGF-1 (outside puberty and pregnancy) in two measurements, but GH < 0.4 µg/L in the OGTT, were selected. Five years after initial evaluation, the patients were submitted to clinical and laboratory (serum IGF-1) reassessment. Patients with persistently elevated IGF-1 were submitted to a new GH suppression test and magnetic resonance imaging (MRI) of the pituitary.

**Results:**

Four patients were lost to follow-up. During reassessment, 42 patients continued to show no “typical phenotype” or changes in physiognomy. Fifteen of the 42 patients had normal IGF-1. Among the 27 patients with persistently elevated IGF-1 and who were submitted to a new OGTT, GH suppression was confirmed in all. Two patients exhibited a lesion suggestive of microadenoma on pituitary MRI. In our interpretation of the results, acromegaly was ruled out in 40 patients and considered “possible” in only 2.

**Conclusion:**

Our results show that even in patients with a suggestive clinical scenario and elevated IGF-1, confirmed in a second measurement and without apparent cause, acromegaly is very unlikely in the case of GH suppression in the OGTT.

## INTRODUCTION

As emphasized by some authors, acromegaly is not always accompanied by a typical phenotype: “acromegaly is a clinical syndrome that may not manifest with clear diagnostic features” (1), “some patients with acromegaly have mild or absent clinical features” (2), “the diagnosis does not require the presence of typical phenotypic features” (3), and “we suggest the measurement of IGF-1 in patients without the typical manifestations of acromegaly, but who have several associated conditions” (4). Therefore, patients with a suggestive clinical scenario should be investigated even in the absence of typical phenotypic features (1-5). The finding of elevated IGF-1 outside puberty and pregnancy strongly supports the hypothesis of acromegaly. Although the diagnosis is confirmed traditionally by the lack of GH suppression in the oral glucose tolerance test (OGTT), cases of acromegaly in the presence of nadir GH < 1 µg/L (6-9) and even < 0.4 µg/L (8,9) have been reported. Many authors therefore consider that the disease should not be readily excluded based on GH suppression in patients with a suggestive clinical scenario and elevated IGF-1 (2,8-12). “Recognition that acromegaly can be accompanied by apparently normal GH concentrations and dynamics, and mild or absent clinical features indicates the importance of IGF-I measurements for diagnosis” (2).

The objective of the present study was to report the evolution of patients with a suggestive clinical scenario (1-5) and elevated IGF-1 (in two measurements and in the absence of another apparent cause), but GH suppression in the OGTT, in whom acromegaly was not initially excluded (2,8-12).

## SUBJECTS AND METHODS

### Patients

First, 4,350 adults (age between 18 and 70 years, excluding pregnant women and patients with known pituitary disease) underwent acromegaly screening: 2,270 patients with type 2 diabetes mellitus or glucose intolerance (13), 178 patients who reported “enlargement of their extremities” (14), and 1,902 patients with two or more comorbidities related to acromegaly [including arterial hypertension in 1,806 patients (15)]. In patients with elevated IGF-1, a new measurement was obtained and combined with the measurement of GH during an OGTT. For this study, 46 patients with a suggestive clinical scenario (1-5) according to the definition below (1,4,5), who had elevated IGF-1 (outside puberty and pregnancy) in two measurements, but GH < 0.4 µg/L in the OGTT (1,11,12,16), were selected. The study and its respective protocol were approved by the Ethics Committee of our institution.

### Follow-up

For this study (5 years after initial evaluation), the patients were submitted to clinical and laboratory (serum IGF-1) reassessment. The aim of clinical examination was to identify typical phenotypic features (see below) and changes in physiognomy by comparing current photographs with those obtained at the time of initial evaluation. Patients with persistently elevated IGF-1 were submitted to a new GH suppression test and magnetic resonance imaging (MRI) of the pituitary.

### Definitions

A typical acromegalic phenotype was defined i) by an endocrinologist with experience in the disease (P.W.R.); ii) based on ectoscopy, and iii) considering acral enlargement and maxillofacial changes (4).

A suggestive clinical scenario was defined in the presence of two or more comorbidities related to acromegaly according to the Canadian Consensus (5), American Association of Clinical Endocrinologists (1), and Endocrine Society (4). The comorbidities considered were (1,4,5): i) nonspecific chronic headache (for example, migraine and hypertensive headache were not considered); ii) generalized and persistent excessive sweating; iii) diffuse arthralgias associated with some radiologic alteration (17) in the absence of known rheumatological disease (reported by the patient, suspected, or confirmed in the medical record); iv) chronic fatigue not explained by any other underlying disease (among the diagnoses reported by the patient or present in the medical record); v) bilateral paresthesias (Carpal tunnel syndrome); vi) recently diagnosed diabetes mellitus; vii) recently diagnosed arterial hypertension requiring antihypertensive medication.

## METHODS

The samples were collected in the morning after an approximately 10-h fast, with the subject resting for 20 min before and during the OGTT. For the OGTT, GH was measured before and 30, 60, 90 and 120 min after the oral administration of 75 g anhydrous glucose.

GH was measured with a chemiluminescence assay (Immulite, Diagnostic Products Corporation, Los Angeles, CA) with an analytical sensitivity ≤ 0.05 µg/L. The standard provided by the kit was calibrated against the World Health Organization (WHO) 2^nd^ International Standard (IS) 98/574. The results are expressed as µg/L. IGF-1 was also measured with a chemiluminescent assay (Immulite 2000, Diagnostic Products Corporation, Los Angeles, CA) (analytical sensitivity of 25 μg/L) using antibodies highly specific for IGF-1 and standards calibrated against the WHO IS 87/518 preparation and previously established reference values stratified by age based on a sample of 1,000 subjects rigorously selected in the same town where the study was conducted (18). “Functional separation” (acidification followed by saturation with IGF-II) was the technique used to exclude interference from IGF-binding proteins (IGFBPs) (18).

MRI of the pituitary (at 3 tesla) was obtained using gadolinium as contrast agent.

## RESULTS

The study included 24 women and 22 men aged 30 to 60 years (median 45 years) with initial IGF-1 ranging from 1.05 to 1.5 times the upper limit of normal range (ULN) for age (18). Thyroid dysfunction and pregnancy (in premenopausal women) were excluded in all patients on initial assessment and 5 years later.

Four patients were lost to follow-up. During reassessment, 42 patients continued to show no “typical phenotype” or changes in physiognomy.

Fifteen of the 42 patients had normal IGF-1 (confirmed in two measurements); none of the patients had kidney or liver failure, malnutrition, uncontrolled hypothyroidism or used oral estrogen; six patients had diabetes mellitus, but were compensated at the time of IGF-1 measurement. The body mass index change of these patients ranged from – 1.2 to + 2 kg/m^2^ (initial assessment versus 5 years later).

Among the 27 patients with persistently elevated IGF-1 and who were submitted to a new OGTT, GH suppression was confirmed in all. Comparing the final and initial concentrations, none of the patient exhibited a significant increase in IGF-1, i.e., increment > 20% [limit defined based on the variation found in 100 healthy (rigorously selected) subjects in stable conditions, in whom IGF-1 was measured at an interval of 3 months using the same assay as employed in this study (18)]. The last IGF-1 ranged from 1.12 to 1.63 times the ULN, already considering the current age of the patient. IGFBP-3 was also measured in these 27 patients, and was normal in 17 patients and slightly elevated in 10 (ranging from 1.02 to 1.2 times the ULN). Two patients exhibited a lesion suggestive of microadenoma on pituitary MRI (hypointense nodule measuring 4 and 5 mm in diameter and showing no contrast enhancement after the administration of gadolinium). Details of these two patients are shown in Table 1. It should be noted that other hormone hypersecretions were also excluded in these two cases.

**Table 1:** Results of the last evaluation of patients with microadenoma on MRI

Patient	Sex	Age (years)	IGF-1 (x ULN)	IGFBP-3	Nadir GH (µg/L)	MRI	Clinical scenario
1	M	52	1.5	Normal	0.2	Microadenoma	Osteoarthritis, hypertension, dyslipidemia, GI, hyperhidrosis
2	F	50	1.42	Normal	0.3	Microadenoma	Headache, paresthesias, hypertension, GI

ULN: upper limit of normal range; MRI: magnetic resonance imaging; GI: glucose intolerance.

In our interpretation of the results, acromegaly was ruled out in 40/42 patients and considered “possible” in only 2/42 (with persistently elevated IGF-1 and microadenoma detected by MRI, but with GH suppression and without clinical or laboratory progression).

## DISCUSSION

There is consensus that not only patients with typical phenotypic features should be investigated for acromegaly (1-5). The patients included in this study had two or more comorbidities commonly found in “active” acromegaly (1,4,5), and additional criteria were required so that they were considered compatible with this condition (see Methods). Moreover, the age range of the patients (30-60 years) coincides with that of a higher incidence of the disease. Consequently, there was a suggestive clinical scenario justifying investigation for acromegaly (1-5).

Elevated IGF-1 does not always indicate acromegaly, but its specificity increases when measured outside puberty and pregnancy (situations characterized by physiological elevation of this hormone). Furthermore, the results should be confirmed in a subsequent measurement. One cause of falsely elevated IGF-1 are inadequate limits of normality. When defined using an inadequately selected sample or an insufficient number of subjects, the upper limit may be underestimated and, consequently, an individual with normal IGF-1 may be erroneously classified as having elevated IGF-1. In the present study, IGF-1 was considered elevated based on the limits established from a sample of 1,000 subjects from the same town as the patients included in this study, who were selected rigorously (exclusion of interfering conditions and medications and extremes of body mass index) and stratified by decade of life (18) according to current recommendations (16,19,20). Hence, in the present study “elevated IGF-1” refers to the measurement obtained outside puberty and pregnancy, confirmed in two measurements, and based on adequate normative information.

Although theoretically possible, heterophile antibodies are not cited as possible agents that interfere with serum IGF-1 (19,20). Moreover, the only case report in the literature mentioning interference of these antibodies with the Immulite assay inexplicably found a reduction in IGF-1 (21). The assay used does not show cross-reactivity to insulin or IGF-II and is highly specific for IGF-1. Finally, “functional separation” (acidification followed by saturation with IGF-II) was used to exclude interference from IGFBPs. Nevertheless, using this assay, eventual interference from IGFBPs would cause a reduction in IGF-1 (19,20).

Overweight/obese subjects have higher hepatic sensitivity to GH. This fact explains the maintenance of IGF-1 concentrations within the normal range despite the reduced secretion of GH observed in these individuals (22). However, there is no elevation of serum IGF-1 (22). It has also been suggested that genotype d3 of the GH receptor (d3-GHR) increases sensitivity to this hormone (23). This fact may explain, at least in part, the higher concentrations of IGF-1 in some patients with acromegaly (for a given concentration of GH), or the greater increase in IGF-1 seen in some patients during treatment with GH (23). However, to our knowledge, there is no study reporting an association between the presence of d3-GHR and elevated IGF-1 in individuals without acromegaly and not treated with GH.

Despite the suggestive clinical scenario and careful definition of “elevated IGF-1” used in this study (see above), after 5 years none of the patient exhibited phenotypic features or changes in physiognomy and 1/3 had spontaneous normalization of IGF-1. All of the patients with persistently elevated IGF-1 continued to present GH suppression and 93% had no apparent tumor on MRI.

Although we cannot rule out acromegaly in the two patients of this series with adenoma on MRI, we believe it is highly unlikely. In addition to persistent GH suppression, reassessment after 5 years (without any intervention) corroborates this conclusion. Considering the interval between the onset of manifestations and the diagnosis in the presence of a typical phenotype (24,25), the absence of the latter and of changes in physiognomy after several years makes the disease unlikely. The lack of an increase in IGF-1 after this period also weakens the diagnosis. We therefore believe that the combination of these findings (persistent suppression of GH, absence of the occurrence of phenotypic features or changes in physiognomy and of an increase in IGF-1 after 5 years) renders acromegaly highly unlikely in these two cases.

In a previous study, acromegaly was not diagnosed in any of the adult patients without a clinical suspicion of the disease and with slightly elevated IGF-1 (up to 1.2 x ULN), but this increase was confirmed in only 15% of the patients (a second measurement was unavailable or normal in the remaining patients) (26). Our results now show that even in patients with a suggestive clinical scenario (1-5) and elevated IGF-1 (> 1.2 x ULN in some), confirmed in a second measurement and without apparent cause, acromegaly is very unlikely in the case of GH suppression in the OGTT. Consequently, the indication of pituitary MRI is questionable in this situation. As discussed earlier, known causes of IGF-1 elevation were excluded and analytical interfering agents do not explain the persistently elevated IGF-1 seen in these patients. We do not know whether these individuals correspond to the portion of the “normal” population that exhibits concentrations outside the reference range, are more sensitive to endogenous GH, or have GH hypersecretion, although not tumoral and suppressive in the OGTT. Further studies on this topic are necessary. Additionally, we do not know whether these persistently elevated concentrations of IGF-1 increase the risk of comorbidities despite the absence of acromegaly, remembering that all of these patients had a combination of two or more of these comorbidities.
